# REGULATORS OF COLLAGEN CROSSLINKING IN DEVELOPING AND ADULT TENDONS

**DOI:** 10.22203/eCM.v043a11

**Published:** 2022-04-05

**Authors:** A.J. Ellingson, N.M. Pancheri, N.R. Schiele

**Affiliations:** 1 Chemical and Biological Engineering, University of Idaho, Moscow, ID, USA

**Keywords:** Tendon, development, collagen, crosslinking, advanced glycation end products (AGEs), lysyl oxidase (LOX)

## Abstract

Tendons are collagen-rich musculoskeletal tissues that possess the mechanical strength needed to transfer forces between muscles and bones. The mechanical development and function of tendons are impacted by collagen crosslinks. However, there is a limited understanding of how collagen crosslinking is regulated in tendon during development and aging. Therefore, the objective of the present review was to highlight potential regulators of enzymatic and non-enzymatic collagen crosslinking and how they impact tendon function. The main collagen crosslinking enzymes include lysyl oxidase (LOX) and the lysyl oxidase-like isoforms (LOXL), whereas non-enzymatic crosslinking is mainly mediated by the formation of advanced glycation end products (AGEs). Regulators of the LOX and LOXL enzymes may include mechanical stimuli, mechanotransducive cell signaling pathways, sex hormones, transforming growth factor (TGF)β family, hypoxia, and interactions with intracellular or extracellular proteins. AGE accumulation in tendon is due to diabetic conditions and aging, and can be mediated by diet and mechanical stimuli. The formation of these enzymatic and non-enzymatic collagen crosslinks plays a major role in tendon biomechanics and in the mechanisms of force transfer. A more complete understanding of how enzymatic and non-enzymatic collagen crosslinking is regulated in tendon will better inform tissue engineering and regenerative therapies aimed at restoring the mechanical function of damaged tendons.

## Introduction

Tendon is predominantly composed of collagen, which is hierarchically arranged in fibrils, fibers, and fascicles (Provenzano and Vanderby, 2006). The stretching and sliding of these collagen structures play a role in the ability of tendons to transfer forces between muscles and bones, and ultimately contribute to the strength and deformation mechanisms of tendons (Hijazi *et al*., 2019; Peterson and Szczesny, 2020; Provenzano and Vanderby, 2006; Screen *et al*., 2004; Svensson *et al*., 2013; Szczesny and Elliott, 2014). Collagen crosslinking may be a critical regulator of tendon mechanics and these crosslinks are essential for tendons to withstand physiological forces over an entire lifetime (Heinemeier *et al*., 2013; Thorpe *et al*., 2010b). The mechanisms that regulate the formation of collagen crosslinking, as well as the types and extent of crosslinking, are beginning to be elucidated. As collagen crosslinking impacts both tendon development and aging, an improved understanding of the regulators of crosslinking has the potential to significantly impact strategies focused on tendon tissue engineering, maintenance of tendon function, and healing.

The interfibrillar and intrafibrillar crosslinks between collagen molecules are formed by both enzymatic and non-enzymatic processes, which contribute in a unique way to tendon functions and changes over time. Collagen crosslinking that occurs during embryonic tendon development appears to be mainly regulated by enzymatic processes, such as by LOX activity (Marturano *et al*., 2013; 2014). In collagen, LOX enzymatically catalyzes the reaction of Hyl and lysine residues into reactive aldehyde species, which subsequently spontaneously link to aldehyde species on adjacent molecules (Lucero and Kagan, 2006; Pinnel and Martin, 1958). These LOX-mediated crosslinks lead to the formation of trivalent and divalent crosslinks between collagen molecules.

Non-enzymatic crosslinks, such as AGEs (with glucosepane being a predominant AGE), are spontaneously formed through glycation processes and yield divalent crosslinks between neighboring fibril residues (Sajithlal *et al*., 1998). AGE accumulation is associated with aging and diseases, such as diabetes, and may contribute to increased brittleness as well as limited collagen remodeling. Together, enzymatic and non-enzymatic collagen crosslinks play an important role in elaboration of tendon mechanical properties during development and aging by reinforcing the collagen matrix and possibly facilitating the transfer of mechanical forces throughout the tissue (Gerriets *et al*., 1993).

The effects of enzymatic and non-enzymatic collagen crosslinks on tendon mechanical properties have been explored in several recent reviews (Eekhoff *et al*., 2018; Gjaltema and Bank, 2017; Snedeker and Gautieri, 2014; van der Slot *et al*., 2003). Briefly, both enzymatic and non-enzymatic crosslinks affect the collagen structure, altering both tissue biomechanics (*e.g*., altered tissue elastic modulus, ultimate tensile strength, stress relaxation, *etc*.) and failure mechanisms (*e.g*., crosslink breakage, load transfer, *etc*.). However, future work will need to directly compare how enzymatic *vs*. non-enzymatic crosslinks impact tendon mechanical properties. Despite the important and complex role collagen crosslinking plays in tendon formation and pathology, it is less clear how these crosslinks are regulated. A thorough understanding of the regulation of enzymatic and non-enzymatic crosslinks is needed to better determine how tendon mechanical properties are regulated, to guide the development of mechanically functional engineered tendon tissues, and to better characterize tendon pathogenesis. Further, manipulation of crosslinks may be applied to a variety of tissue engineering techniques that use collagen-based biomaterials to modulate mechanical properties and failure mechanisms. Therefore, the present review aimed to highlight recent findings on the potential regulators of enzymatic and non-enzymatic collagen crosslinks in tendon, as well as to discuss the limited knowledge on collagen crosslinking in tendon development, disease, and aging. To further develop novel effective treatments for damaged tendons, an improved understanding of collagen crosslinks and the mechanisms governing their formation is needed.

## Collagen fibrillogenesis and role of enzymatic crosslinking

### Intracellular fibrillogenesis

Embryonic tendon is characterized by a transition from a dense cellular network, with relatively minimal collagen content, to a structure defined by an organized collagen matrix (Richardson *et al*., 2007; Schiele *et al*., 2013; Schiele *et al*., 2015; Theodossiou *et al*., 2020). This process of collagen fibrillogenesis has been previously described (Banos *et al*., 2008; Bayer *et al*., 2010; Canty *et al*., 2006; Canty and Kadler, 2005; Kadler *et al*., 2008) but will be briefly reviewed along with the formation of the two primary forms of mature enzymatic crosslinks, HP and LP, in tendon.

**Fig. 1.**
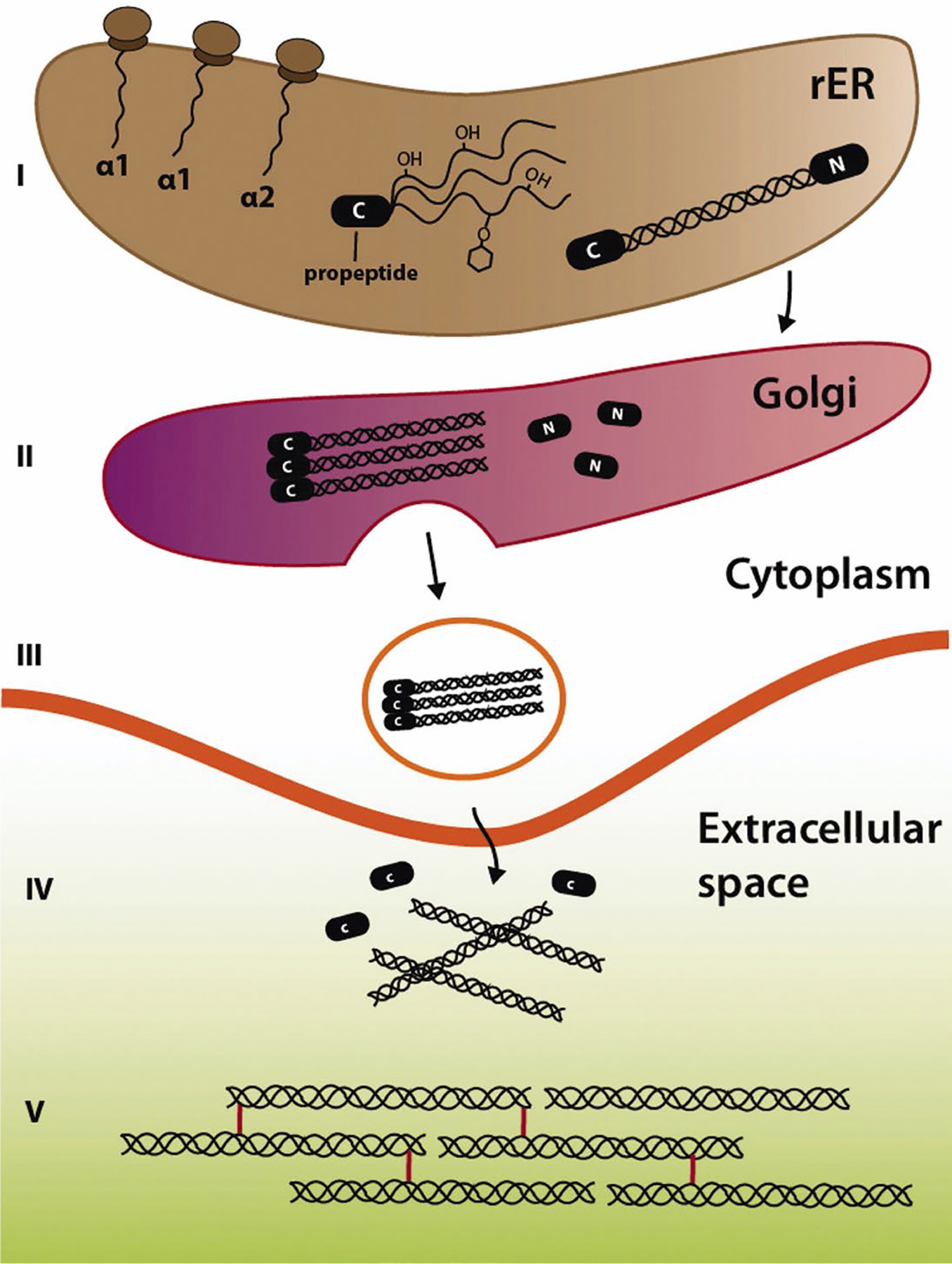
Summary of collagen fibrillogenesis. (I) Fibrillogenesis begins with production of procollagen-α-chains (α1–3) and their aggregation into collagen pro-peptides in the rough ER (rER). (II) Pro-peptides are processed and packaged in the Golgi apparatus before being secreted into the (III) extracellular space for maturation and crosslinking, (IV) including cleavage of remaining N-terminal (N) and C-terminal (C) domains to produce (V) mature tropocollagen. Reprinted with permission from Gjaltema and Bank (2017).

Collagen fibrillogenesis begins with the translation of collagen pre-pro-α-polypeptide chains, which subsequently undergo hydroxylation of proline and lysine residues. Lysine hydroxylation is mediated by the enzyme LH. Consisting of three distinct isoforms, LH1 primarily hydroxylates lysine along the helical domain of the α-chains to form Hyl (Gjaltema and Bank, 2017). Together, glycosylation of Hyl residues, hydroxylation of proline *via* the complex P4HA/PDI and P3H isoforms, and formation of disulfide bonds initiate aggregation of α-chains into a triple helix to form a procollagen molecule that is secreted by a collagen-producing cell into the ECM through a vesicle. This secreted molecule is cleaved at the N- and C-terminal by procollagen peptidases to produce mature tropocollagen (Gjaltema and Bank, 2017) (Fig. 1). Remaining telopeptide lysine residues can be hydroxylated through LH isoforms, possibly LH2 (Gjaltema and Bank, 2017; van der Slot *et al*., 2003). Notably, LH1 and LH2 (consisting of the splicing isoform LH2a and LH2b) are primarily associated with the major collagen types (I, II, and III), whereas LH3 is primarily associated with the minor collagen types (*e.g*., IV and V) (Gjaltema and Bank, 2017; Risteli *et al*., 2004; van der Slot *et al*., 2003). LH1 primarily hydroxylates lysines in the helical domain of procollagen, whereas LH2 functions within the telopeptidyl domain (Myllylä *et al*., 2007; Takaluoma *et al*., 2007; van der Slot *et al*., 2003). In a mouse development model, all LH isoforms were found expressed at various levels during embryogenesis and in a tissue-dependent manner (Salo *et al*., 2006). Specifically, all LH isoforms were expressed in the murine mesoderm at embryonic day (E)7.5 and had continued expression in later mesoderm-derived tissues. LH1 is highly expressed throughout embryonic development of mice within collagen type I, II, and III. LH2a is expressed in whole mouse embryos until E11.5 and, then, localizes to the brain, kidney, and testis (Salo *et al*., 2006; Walker *et al*., 2005). LH2b (long splice form) expression begins at E11.5 and is the primary LH isoform found throughout adulthood in muscle, lung, and connective tissue. LH3 appears to have a more housekeeping function (Salo *et al*., 2006).

### Extracellular fibrillogenesis

**Fig. 2.**
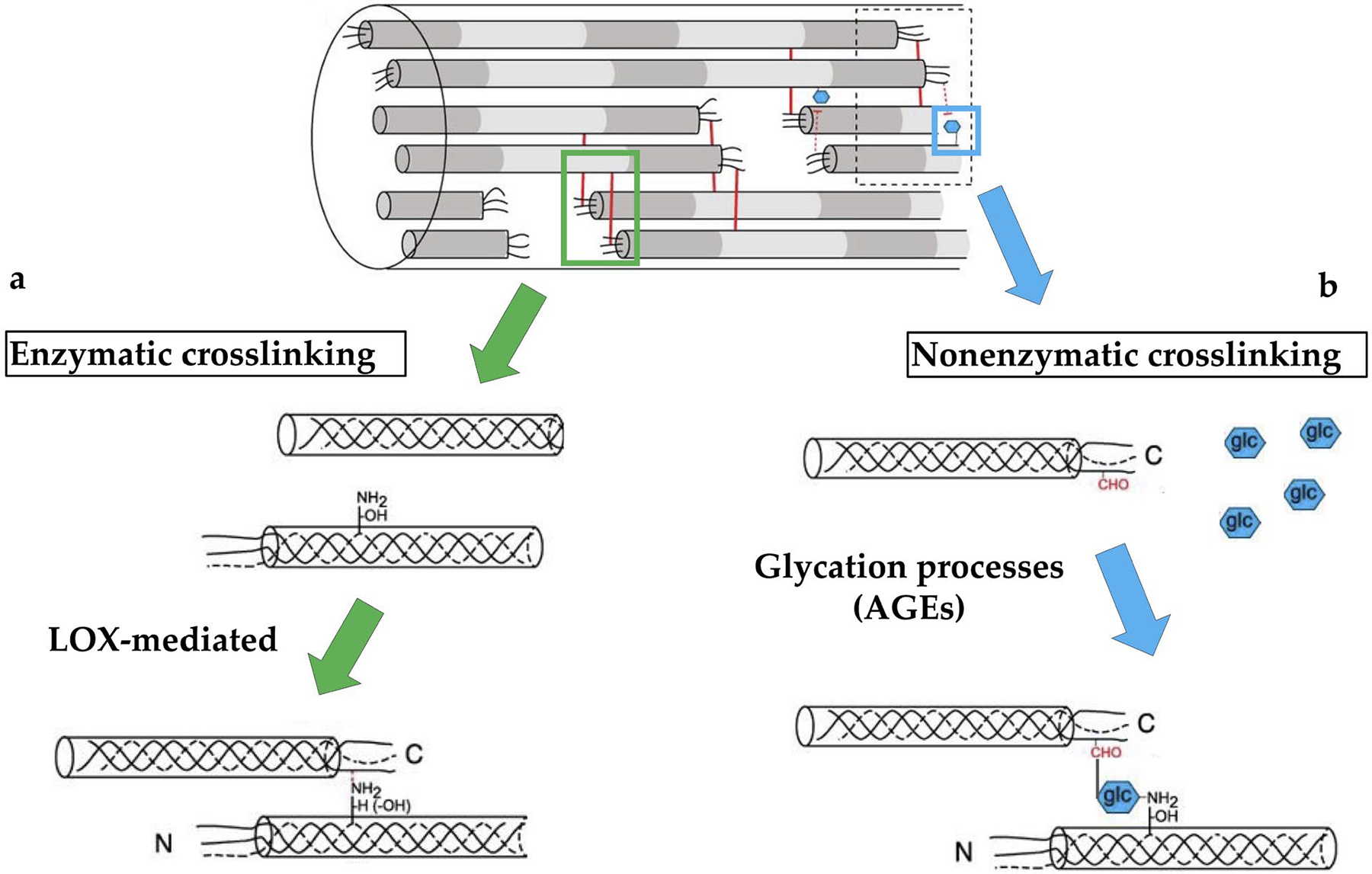
Enzymatic *versus* non-enzymatic processes. The two primary forms of crosslinks in mature tendon collagen can be identified as being driven by enzyme activity (*i.e*., enzymatic crosslinks) or other processes (*i.e*., non-enzymatic). (**a**) Enzymatic crosslinks are primarily mediated by LOX, which catalyzes the conversion of lysine residues (NH_2_) into reactive aldehyde species (-H) and yield immature or mature divalent and trivalent crosslinks. (**b**) The dominant non-enzymatic crosslinks are AGEs, which are formed through glycation of protein residues, such as lysine (NH_2_), by reducing sugars, such as glucose (glc). Modified with permission from Hudson *et al*. (2018).

**Fig. 3.**
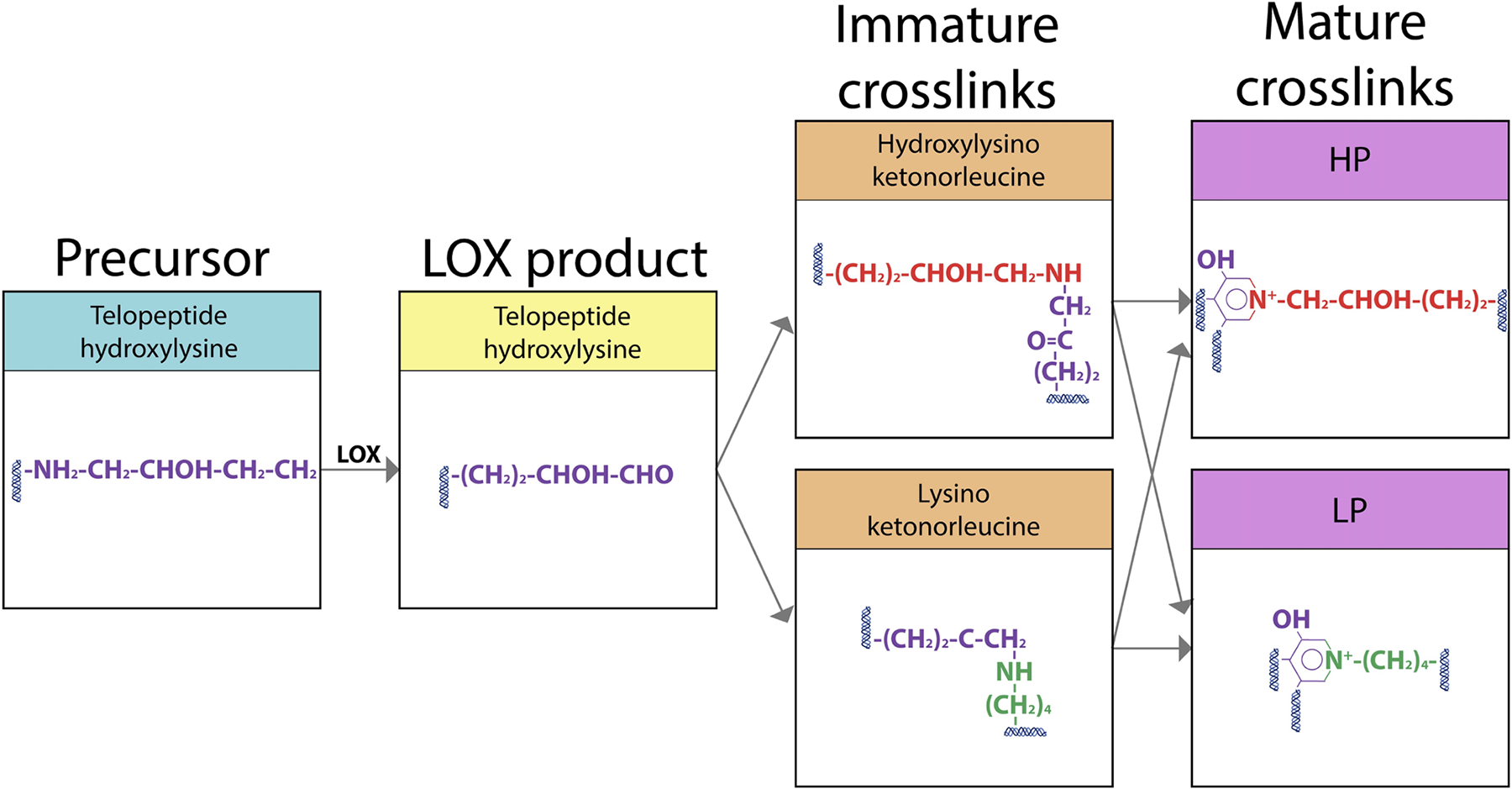
LOX-mediated collagen crosslinking. LOX-mediated formation of divalent and trivalent crosslinks in collagen fibrils in tendons. Modified with permission from Makris *et al*. (2013).

Following secretion and hydroxylation of the triple-helical procollagen, LOX catalyzes the formation of Hyl and lysine residues into reactive aldehyde species. In turn, these species spontaneously link to aldehydes on adjacent collagen molecules by covalent bonds to form immature divalent crosslinks (Fig. 2a). Immature divalent crosslinks may subsequently link with further residues on another telopeptidyl residue to create a single mature trivalent crosslink (Eyre *et al*., 1984; 2008). The most abundant types of enzyme-mediated crosslinks within adult tendon are mature trivalent HP (HP or HylPyr, or pyridinoline, 428 Da) crosslinks – composed of two telopeptide Hyl and one helix Hyl – and, to a lesser extent, trivalent LP (LP or LysPyr, or deoxypyridinoline, 412 Da) crosslinks – composed of two telopeptide Hyl and one helix lysine (Fig. 3) (Eyre *et al*., 1984; Eyre *et al*., 2008).

A recent study has suggested that these LOX-mediated crosslinks are formed at the fibril surface during fibrillogenesis because the LOX molecule (3.7 nm radius) may be too large to diffuse within the collagen fibril (Leighton *et al*., 2021). While unknown in tendon, previous work in bone has identified the gap spacing (*i.e*., porosity) between the tropocollagen molecules within a collagen fibril to be around 2 nm (Xu *et al*., 2020), thereby inhibiting LOX diffusion. Furthermore, the molecular weight of LOX (32 kDa) approaches the upper limits of collagen type I pore size, estimated to be between 5.7 kDa and 48 kDa, which may limit LOX ability to diffuse into the fibril (Toroian *et al*., 2007). Differences in HP and LP forms (*e.g*., Hyl *vs*. lysine) are driven by LH1 and LH2 during fibrillogenesis (Hudson *et al*., 2017; Terajima *et al*., 2016). However, the specific mechanisms governing LH-mediated processes are not well understood in tendons or ligaments. Other types of enzymatic crosslinking are more or less prevalent among different types of tendons, such as Hyl pyrrole and lysyl pyrrole (Eekhoff *et al*., 2018; Thorpe *et al*., 2010a).

While LOX appears to be the primary mediator of enzymatic collagen crosslinking in tendon, other enzymes, such as TGs, may also impact ECM crosslinking (Taye *et al*., 2020) as well as cell behavior and tissue pathologies (Eckert *et al*., 2014; Lorand and Graham, 2003). Recently, TG2 was found in the myotendinous junction of 5-month old mice (Jacobson *et al*., 2020). TGs have been identified in human articular cartilage (Summey *et al*., 2002) and may play a role in supraspinatus tendon pathogenesis (Oliva *et al*., 2009). The specific effects of TGs and similar enzymes on tendon formation remain largely unknown and need further study. Therefore, the present review will focused on LOX-mediated crosslinks in tendon development and aging.

## Crosslinks identified in mature tendon

**Fig. 4.**
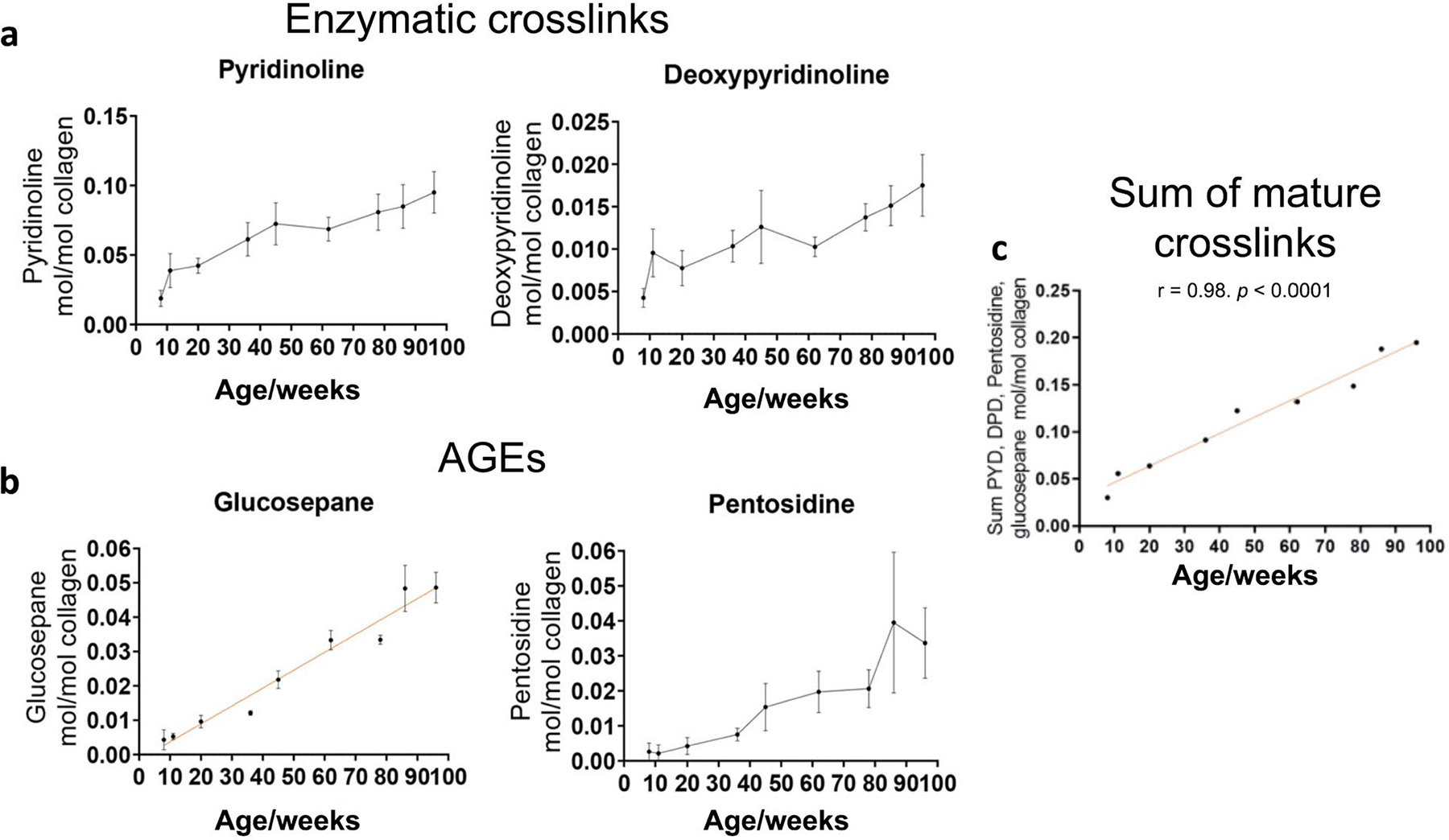
Collagen crosslinking changes over time in mouse tail tendon. (**a**) Mature enzymatic crosslinks, HP and LP, increase with aging. (**b**) AGEs (glucosepane and pentosidine) and (**c**) total mature crosslinks increase with aging. Modified with permission from Stammers *et al*. (2020a).

Concentrations of HP and LP crosslinks appear to increase over time throughout maturation in mouse tail tendon (Stammers *et al*., 2020a) (Fig. 4a). Functionally distinct tendons contain different amounts of mature trivalent crosslinks (Carroll *et al*., 2012; Svensson *et al*., 2013; Svensson *et al*., 2018). For example, in 16-week-old rats, HP crosslink content was 139-fold higher in Achilles tendons, an energy-storing tendon, compared to tail tendons, a positional tendon, whereas LP crosslink content was 1.6-fold higher in tail tendons (Svensson *et al*., 2018). Furthermore, flexor and extensor tendons have different crosslink types and densities, with flexor tendons containing more thermally stable crosslinks (*i.e*., more mature crosslinks) and a higher crosslink density (Herod *et al*., 2016). Despite this, extensor tendons are significantly stronger and tougher than flexor tendons (Herod *et al*., 2016). The role of crosslinking and its contribution to these mechanical properties requires further investigation, but it is proposed that a trade-off between strength and fatigue resistance and crosslinking may play a role in the mechanical differences between tendon types.

Crosslinking may also differ between mature tendons and ligaments. Comparing human cadaveric ACL, patellar tendon, and semitendinosus tendon, ACLs were found to have a larger total number of enzymatic crosslinks than either tendons and no differences were observed between patellar tendon and semitendinosus tendon (Suzuki *et al*., 2008). Similarly, cells derived from rabbit ACL, MCL, and patellar tendon and cultured for 4 weeks showed distinct biochemical profiles (Kato *et al*., 2015). ACL-derived fibroblasts had significantly larger total enzymatic crosslinking content (total divalent crosslinks plus pyridinoline crosslinks) and increased *PLOD1/2*, *LOX*, *Col1a1*, and *Col3a1* expression, compared to MCL-and patellar-tendon-derived fibroblasts, with no differences between MCL and patellar tendon cells (Kato *et al*., 2015). These studies suggest that functionally distinct ligaments and tendons have unique crosslink maturation processes (Kato *et al*., 2015; Suzuki *et al*., 2008). However, the mechanisms governing these maturation processes are not well characterized.

Equine SDFTs are commonly used as a functional equivalent to human Achilles tendon (Patterson-Kane and Rich, 2014) and they are the most commonly injured tendon in horses (Thorpe *et al*., 2010a). Using HPLC, a positive correlation between pyrrole crosslinking densities and mechanical properties, including elastic modulus, ultimate stress, and yield stress, was identified in SDFTs. However, the predominant crosslink, HP, did not correlate with mechanical function (Thorpe *et al*., 2010a). Similar work in human patellar tendon corroborated the finding that HP and LP crosslinks are likely to only play a minor role in tissue mechanics after initial tissue formation (Svensson *et al*., 2013).

The different crosslinks and concentrations within various tendon types, particularly tendons of different functions (*i.e*., positional *vs*. energy-storing tendons), raise intriguing questions regarding the specific role of enzymatic crosslinks in these tissues and how they are differentially regulated.

### Crosslinks at the tendon-bone interface

The mechanical properties and structure of tendon change spatially as the tissue transitions from the tendon itself (mainly collagen) to its insertion site into the bone (mainly mineralized collagen). The tendon-bone interface (enthesis) is particularly pertinent because partial- and full-thickness tendon tears at the enthesis are a primary cause of rotator cuff injury (Spargoli, 2016; Yamamoto *et al*., 2010) and typically require a surgical repair. The enthesis demonstrates a lower modulus than other regions of the tendon, possibly leading to increased deformation and energy absorption prior to failure, creating an overall tougher tissue (Deymier *et al*., 2017). Mineral content, collagen orientation, and protein gradients all vary throughout the length of the enthesis (Deymier-Black *et al*., 2015; Thomopoulos *et al*., 2003). Specifically, closer to the tendon side, more aligned collagen fibers along with higher concentrations of decorin and biglycan are observed (Deymier-Black *et al*., 2015). As the collagen fibers in tendon becomes ossified (transition to bone), collagen becomes less aligned and localized aggrecan (a proteoglycan localized in other regions of the tendon that experience high compressive forces) content becomes prominent (Thomopoulos *et al*., 2003; Waggett *et al*., 1998). A significant knowledge gap exists in the heterogeneity of collagen crosslinking and associated mechanisms (including types of crosslinks, LOX, LH, *etc*.), particularly within critical transition regions of the enthesis. A better understanding of these phenomena may aid in the development of tissue-engineered constructs and regenerative therapies targeted at treating evulsion injuries.

## LOX production and activation

The copper-dependent amine oxidase LOX, coded by *LOX*, is first transcribed as a pre-pro-protein. Pre-proLOX undergoes post-translational modifications within the ER and, then, experiences N-glycosylation of the N-terminal pro-peptide chain (147 aa residues) and folding of the C-terminal (containing the mature protein, 249 aa residues) to form three or more disulfide bonds (Trackman *et al*., 1992; Williams and Kagan, 1985). Then, proLOX is secreted into the ECM where the glycosylated N-terminal is proteolytically cleaved by BMP-1, also known as PCP, to release the active mature LOX protein and generate a LOX-PP (Cronshaw *et al*., 1995; Maruhashi *et al*., 2010; Uzel *et al*., 2001). The mechanisms regulating BMP-1 in tendon are not currently well known but, considering the role it plays in LOX activation, it would be worth investigating them. LOX-PP may help govern LOX activity by mediating secretion of proLOX into the ECM, and glycosylation of LOX-PP aids in efficient protein folding (Grimsby *et al*., 2010). To the authors’ knowledge, the specific role of LOX-PP in tendon has not been characterized and further investigation could improve the understanding of LOX regulation. Notably, LOX incorporates a copper cofactor and an organic peptidyl cofactor (LTQ) that are needed for proper function (Gacheru *et al*., 1990; Wang *et al*., 1996). As a copper-dependent enzyme, the addition of copper sulfate to cell culture increases the total HP crosslinks and tensile properties (which can be further enhanced by treatment with exogenous Hyl) in engineered neocartilage constructs through enhanced LOX activity (Makris *et al*., 2013).

### LOXL isoforms

The LOX superfamily consists also of 4 additional isoforms, LOXL1-4. A copper-binding domain, LTQ, and a CRL domain on the C-terminal are conserved through all 5 paralogues (Lee and Kim, 2006). LOXL1, while the most morphologically similar to LOX, appears to primarily target elastin and tissues undergoing elastogenic events, making it less relevant to tendon-collagen crosslinking (Liu *et al*., 2004; Zhao and Zhou, 2012). Furthermore, elastin deficiency has minor impacts on murine Achilles tendon mechanical properties (Eekhoff *et al*., 2017), suggesting that LOXL1 may have a minimal role in tendon formation. However, this needs further study. Whereas LOX and LOXL1 both require proteolytic cleavage for activation, LOXL2-4 are active in both processed and non-processed forms (Rodriguez *et al*., 2010; Trackman *et al*., 1992). LOXL2 and LOXL3 isoforms exhibit amine oxidase activity towards collagen and elastin (Kim *et al*., 2011; Lee and Kim, 2006). Although not well explored in tendon formation, knockdown of LOXL2 in chondrogenic cell lines blocks further chondrogenesis through increases in SNAIL, a transcriptional repressor of cadherins, and decreases in SOX9, a transcription factor and key regulator of chondrogenesis (Iftikhar *et al*., 2011). Application of exogenous LOXL2 to cultured cartilage explants (derived from bovine articular cartilage) and engineered chondrogenic constructs (primary chondrocytes cultured to create a self-assembled structure), which were later implanted into athymic mice, promoted tissue maturation (increased mature crosslinks) and enhanced tensile mechanical properties (Makris *et al*., 2014). LOXL3 appears to have a critical role in facilitating development of the myotendinous junction and proper formation of the fibronectin matrix within mouse embryos (Kraft-Sheleg *et al*., 2016). Increases in LOXL2-4 correlate with fibrotic conditions in skin and lung, often identified by abnormal stiffness through excessive ECM and collagen deposition and crosslinking. However, this needs further investigation in tendon (Huang *et al*., 2019; Huang *et al*., 2020; Jones *et al*., 2018). Together, the impact of LOX-like protein isoforms on tissue development justifies additional investigation for applications in tendon tissue engineering.

### Impacts of LOX inhibition on tendon development

Complete suppression of *LOX* results in perinatal murine death due to compromised collagen networks in the cardiovascular system and in abnormal collagen fiber morphology and organization within the dermis (Hornstra *et al*., 2003; Mäki *et al*., 2002; Mäki *et al*., 2005). No known animal models have been developed that allow for selective activation or inactivation of *LOX* during tendon development or in adulthood. Such a model would be beneficial to studying how LOX regulates tissue formation, homeostasis, and healing. However, to investigate LOX, current techniques rely mainly on the use of various ligands to inhibit its activity. These inhibitors act through two mechanisms: i) interaction with the copper-dependent cofactor (*e.g*., thiram, disulfiram, MCP); ii) interaction with the LTQ prosthetic group at the LOX active site (*e.g*., BAPN, TCP) (Hajdú *et al*., 2018).

BAPN is the most widespread irreversible inhibitor of LOX used in tendon studies. Although BAPN is broadly recognized as a standard in the tendon field, evidence suggests that BAPN treatment is not effective against LOXL2 (Kim *et al*., 2011); while LOXL3 is sensitive to inhibition by BAPN treatment (Kim *et al*., 2011; Lee and Kim, 2006). Others point to BAPN as an inhibitor of the entire LOX superfamily due to its affinity for amine oxidases (Rodríguez *et al*., 2008; Tang *et al*., 1983). Few studies have focused upon elucidating the specific effect of BAPN on individual LOX isoforms and more work should be conducted on how these inhibitory molecules specifically interact within the tendon. As such, interpretations of current studies may be limited by a lack of understanding on the specificity of BAPN inhibition. Specifically, conclusions regarding the effects of LOX using BAPN may not directly consider the potential activity of LOXL2-4 within the tendon. However, BAPN has been a powerful tool in determining how enzymatic collagen crosslinking impacts embryonic tendon formation.

Several studies using BAPN in a chick model of embryonic tendon formation have shown that LOX is essential for increased elastic modulus during development (Marturano *et al*., 2013; Marturano *et al*., 2014; Pan *et al*., 2018). Specifically, treatment of embryonic chick with varying concentrations of BAPN (5 mg/g or 15 mg/g) results in a decreased nanoscale elastic modulus of the calcaneal tendon at HH 40 and HH 43 without identified changes to GAG/dry mass, hydroxyproline/dry mass, cell viability, or cell density (Marturano *et al*., 2013). A higher concentration of BAPN (15 mg/g) results in a greater decrease in elastic modulus than a lower concentration (5 mg/g). BAPN also decreases the ratio of HP/collagen and LP/collagen, compared to controls (Marturano *et al*., 2014). The HP/LP ratio remains constant, suggesting BAPN acts on enzymatic collagen crosslinking, but without substrate preference towards HP or LP crosslinks (Marturano *et al*., 2014). In a different study of chick calcaneal tendon, *LOX* expression changed between HH 38 and HH 45, with peak expression at HH 42. LOX activity steadily increased over these same time points (Pan *et al*., 2018). Interestingly, proLOX activity peaked at HH 43, which is when chick embryos exhibit peak motility. Each LOX isoform exhibited a distinct gene expression profile. *LOXL2* and *LOXL4* decreased at HH 41, 42, 45 and HH 39–45, respectively, relative to HH 38, but *LOXL1* and *LOXL3* remained relatively constant, emphasizing each isoform may have a distinct role in embryonic tendon development (Pan *et al*., 2018).

Less work on LOX has been performed in human or mammalian tendon models. However, one study used an *in vitro* tendon-construct model that incorporated adult human tendon-derived fibroblasts in a fibrin gel (Herchenhan *et al*., 2015). Constructs immediately treated with BAPN (day 0) were untestable and resulted in a ruptured construct. Constructs first cultured for 14 d, and then treated with BAPN, showed increased collagen type I monomers and dimers compared to controls at 21 d of culture (*i.e*., less crosslinked). BAPN treatment resulted in a mechanically weaker construct (decreased failure stress, failure strain, and tensile modulus) and abnormal collagen fibrils that resembled Ehlers-Danlos collagen phenotypes (Herchenhan *et al*., 2015). BAPN treatment did not appear to affect *Col1a1* expression or collagen type V, decorin, fibromodulin, or tenascin-X.

Based on these studies, LOX appears to be an important regulator in the formation of tendon mechanical properties. However, separating the role of collagen crosslinking from larger structural changes to collagen over time remains a challenge. Specifically, collagen organization (*i.e*., fibril overlap and elongation, interweaving, bifurcation/fusion, *etc*.) changes throughout tendon development, demonstrating greater structural order (*i.e*., fewer bifurcations and less interweaving) in mature compared to fetal tendons (Provenzano and Vanderby, 2006). Further, some of these interweaving collagen fibrils may be retained in mature tendon and their contributions to mechanical function are being evaluated, with evidence that friction between helical fibrils transfers mechanical loads (Safa *et al*., 2019; Szczesny *et al*., 2017). Overall, further work is needed in mammalian systems to identify specifically how LOX and LOXL1-4 are regulated during tendon development, as well as better differentiate between changes to collagen crosslinking and the broader changes to ECM structure.

## Potential regulators of enzymatic crosslinking

**Fig. 5.**
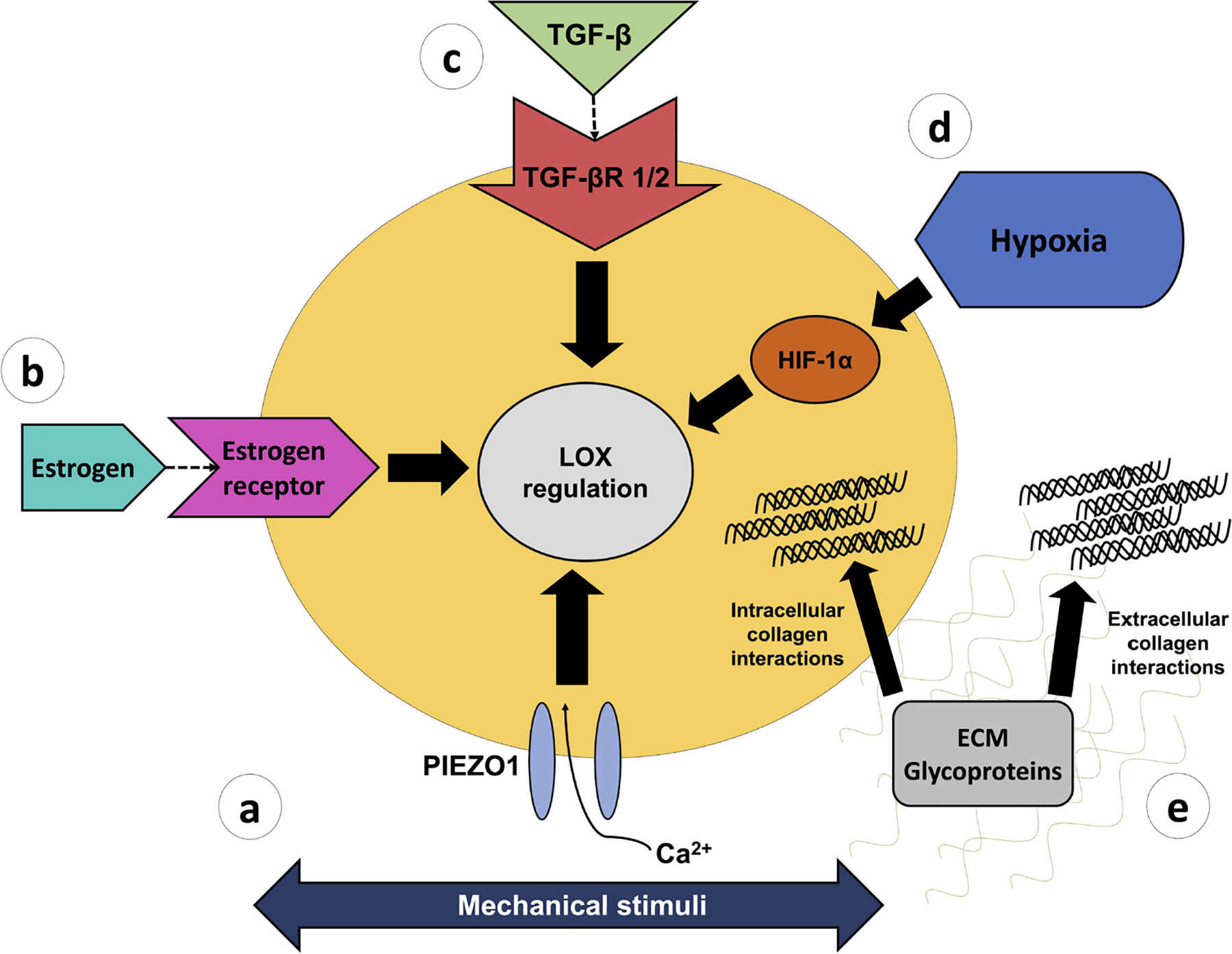
Potential cell-level regulators of enzymatic crosslinking in tendon. Enzymatic crosslinking, mediated by LOX, may be regulated by (**a**) mechanoresponsive signaling pathways, such as the calcium ion channel PIEZO1, (**b**) signaling through the sex hormone estrogen, (**c**) TGFβ family, (**d**) hypoxia-induced HIF-1α signaling, and (**e**) intracellular and extracellular collagen interactions with glycoproteins including fibromodulin and TSP-1.

Enzymatic crosslinking mediated by LOX appears to play an important role in the mechanical formation of tendon. Therefore, knowing the mechanisms that regulate LOX production and activity is needed to understand and ultimately regulate functional tendon development. While there is limited information on specific cell signaling pathways or external factors that contribute to LOX regulation at the cellular level in tendon, potential regulators that will be discussed include mechanical stimuli, hormone interactions, growth factors, hypoxia, and various glycoproteins in the ECM (Table 1, Fig. 5).

### Mechanical stimuli

Tendons experience mechanical stimulation from muscle contractions. The impact of muscle contractions on tendon and LOX has been explored during embryonic chick development (Pan *et al*., 2018). Chick embryos exhibiting a hypermotile (*i.e*., excessive mechanical loading) profile (through treatment with 4-aminopyridine) show an increased calcaneal tendon elastic modulus relative to controls, without changes in *LOX* expression. However, chick embryos treated with rigid and flaccid paralysis (unloading) phenotypes (through treatment with decamethonium bromide or pancuronium bromide) have a decreased calcaneal tendon elastic modulus that corresponds to decreased LOX activity. Interestingly, a hypermotility plus BAPN treatment does not significantly alter the elastic modulus (Pan *et al*., 2018). Together, these findings suggest that mechanical stimuli from muscle contractions during embryonic development impact tendon modulus through alterations to LOX production and, therefore, collagen crosslinking.

A recent study using rat tendon explants and genetic manipulations in mice showed that adult tendon cells may detect some modes of mechanical loading through the mechanosensitive ion channel PIEZO1 (Passini *et al*., 2021). Diminished PIEZO1 activity decreases stiffness, whereas enhanced PIEZO1 activity increases stiffness of rat tail tendons. Furthermore, pharmacologically induced PIEZO1 activation (through Yoda1), combined with mechanical stretch, upregulates *Lox* expression in tendon fascicle explants. Mice with a PIEZO1 gain-of-function mutation display increased crosslink density, as indicated by thermal calorimetry and fluorescence imaging, and with no apparent changes to fibril structure or organization (Passini *et al*., 2021). These results may suggest that enzymatic collagen crosslinking is at least partially governed by the ion channel PIEZO1 and by mechanical stimuli. Further research into LOX activation by PIEZO1 and how and which mechanosensing pathways are affected by mechanical stimulation is necessary to help the understanding of LOX regulation and collagen crosslinking by tendon cells.

#### Mechanoregulation in PDL

hPDL cells embedded within a collagen gel respond uniquely to different magnitudes of mechanical loading. Low tensile strain magnitudes (3 %) upregulate *LOX*, *Col1a1* and *Col3a1* expression, LOX activity, and production of secreted collagen (Chen *et al*., 2013). In contrast, high tensile strain magnitudes (10 %) do not affect LOX but downregulate *Col1a1* and upregulate *Col3a1*, *MMP2*, and *TIMP2* expression (Chen *et al*., 2013). One potential explanation for these phenomena is that low level mechanical stimulation (3 % strain) facilitates ECM deposition and stabilization *via* LOX-mediated collagen crosslinking, whereas high level stimulation (10 % strain) favors ECM degradation and tissue remodeling (Chen *et al*., 2013). A similar study on hPDL cells applying varying magnitudes of compressive mechanical loading found *LOX* and *LH2* expression increased at low but decreased at high loading magnitudes (Kaku *et al*., 2016). This same study explored the *in vivo* effects of loading in rats using an excessive occlusal loading model (*i.e*., a steel wire was added to the left first molar to induce excessive contact between teeth). Excessive loading increased collagen maturation as well as LOX and LH2 positive cells. Excessive loading plus BAPN treatment suppressed the relative increases in LOX and LH2 positive cells, as well as collagen maturation (Kaku *et al*., 2016). This suggests that PDL collagen maturation in an excessive loading model is at least partially driven by mechanosensitive enzymatic crosslinker activity. Together, these studies on the mechanosensitive behavior of PDL tissue and cells justifies further investigation in tendon tissue, specifically on the impacts of varying magnitudes of mechanical load and the correlation between ECM remodeling (*e.g*., MMPs) and stabilizing (*e.g*., LOX) proteins.

#### Positional tendons versus energy-storing tendons

Further evidence for a mechanosensitive crosslinking-driven mechanism comes from the different types of crosslinks identified in positional *versus* energy-storing tendons (Carroll *et al*., 2012; Svensson *et al*., 2013; Svensson *et al*., 2018). A possible explanation for the disparate crosslinking quantities are the functional differences between these tendon types and the different loading magnitudes. Energy-storing and weight-bearing tendons, such as the Achilles tendon, are repeatedly exposed to a greater mechanical load than tail tendons, suggesting LOX and the resulting crosslinks may be mechanically regulated. However, it is possible that differences between tendon types are not related to LOX regulation and mechanical loading but to underlying differences during formation due to unknown growth factors, signaling, genetic variation, *etc*.. These potential regulators of LOX along with mechanical stimuli should be investigated in future studies.

### Hypoxia

**Fig. 6.**
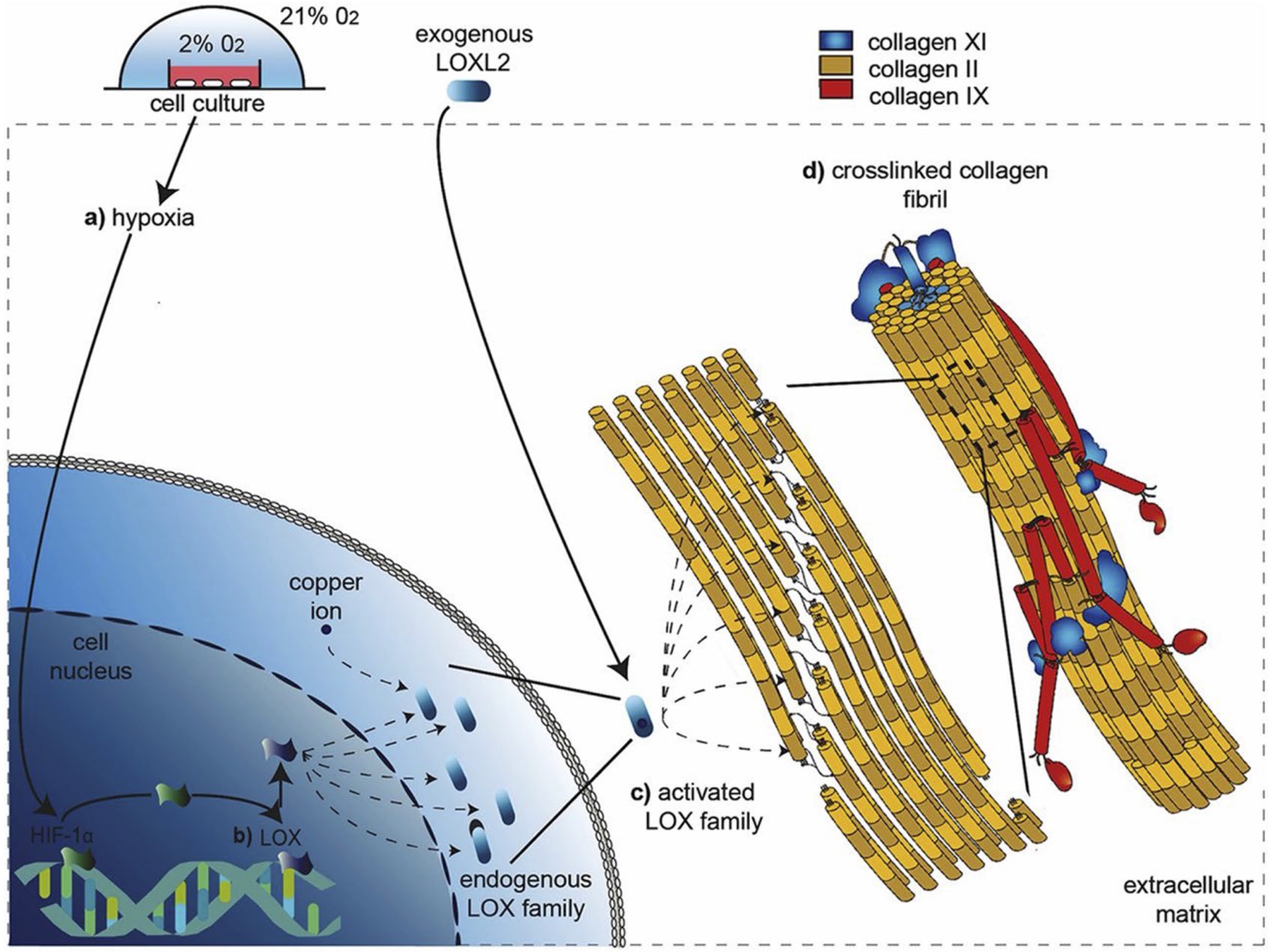
Effect of hypoxia on crosslinking. Hypoxic environments may regulate LOX-mediated collagen crosslinking. (**a**) *In vitro* cell cultures exposed to hypoxia may upregulate LOX production *via* the HIF-1 pathway. (**b**) Promotion of *LOX* and *LOXL* increase activity of respective isoforms (LOX, LOXL1-4). (**c**) LOX and LOXL isoforms drive collagen maturation by catalyzing the formation of intermolecular bonds within and between collagen fibers to form HP and LP crosslinks. (**d**) Fibrillar collagen crosslinks catalyzed by LOX enhance tissue mechanical properties. Reprinted with permission from Makris *et al*. (2014).

The oxygen tension of tendon *in vivo* is not well documented but the minimal vasculature of tendon suggests it is a relatively hypoxic tissue, presumably similar to articular cartilage (1–10 % oxygen) (Grimshaw and Mason, 2000) or bone marrow (1–7 % oxygen) (Antoniou *et al*., 2004; Chow *et al*., 2001; D’Ippolito *et al*., 2006). Low oxygen levels in tendon have the potential to regulate LOX, however literature exploring hypoxia and LOX does not focus primarily on tendons. A hypoxic environment (2 % oxygen) increases *LOX* expression, Young’s modulus, and the concentration of HP crosslinks in bovine articular cartilage explants after 4-week culture (Makris *et al*., 2014) (Fig. 6). When BAPN treatment is used in combination with hypoxia, *LOX* expression increases (although at a level lower than hypoxia treatment alone) but Young’s modulus and HP crosslinks are not impacted, relative to controls. Together, this suggests that hypoxia may mediate *LOX* expression and activity to impact tissue modulus. Hypoxia and BAPN treatments were also applied to bovine ACL, PCL, patellar tendon, and knee meniscus explants; the same trends in modulus were detected, although *LOX* expression was not assessed in these tissues (Makris *et al*., 2014).

Treatment of rat lung fibroblasts with cobalt chloride (a chemical inducer of a hypoxia-mimicking cell response) induces activation of *LOX via* the HIF-1 pathway, specifically, through the hypoxia-response element located in the promoter region of *LOX* (Gao *et al*., 2013). Treatments combining cobalt chloride and cadmium chloride (an inducer of reactive oxygen species) inhibit HIF-1 expression and binding with *LOX* (Gao *et al*., 2013). A study in dermal fibroblasts found that hypoxia promotes collagen deposition *via* HIF-1α and TGFβ/Smad signaling and that HIF-1α knockdown inhibits TGF-β/Smad signaling (Mingyuan *et al*., 2018). Moreover, silencing Smad4 decreases HIF-1α significantly when combined with hypoxia (Mingyuan *et al*., 2018). Furthermore, multiple studies have identified *LH2* expression and LH2 activity to increase under hypoxic conditions (Brinckmann *et al*., 2005; Hofbauer *et al*., 2003; Horino *et al*., 2002; van Vlimmeren *et al*., 2010). Mouse embryonic fibroblasts cultured in a hypoxic environment show a time-dependent 5- to 12-fold transcription up-regulation of *PLOD1* and *PLOD2*, which code for LHs. However, embryonic fibroblasts lacking the HIF-1α subunit are not affected (Hofbauer *et al*., 2003).

While LOX appears to be impacted by hypoxia, the specific cellular pathways are poorly understood, particularly in tendon. Further investigation of this topic has potential to significantly impact the understanding of the hypoxia-dependent mechanisms governing enzymatic crosslinking in tendon.

### TGFβ signaling

TGFβ signaling is a known regulator of tendon differentiation in both embryo and cell culture (Brown *et al*., 2015; Chien *et al*., 2018; Havis *et al*., 2016; Pryce *et al*., 2009; Tan *et al*., 2020; Theodossiou *et al*., 2019; Theodossiou *et al*., 2021). However, while the interactions between the TGFβ family and LOX have been previously investigated in various tissues, they are not well understood in tendon and necessitate further study. TGFβ1 is a potent inducer of enzymatic crosslinks during fibrosis and scar formation (Fushida-Takemura *et al*., 1996; Van Bergen *et al*., 2013). Three members of the TGFβ family (TGFβ1, 2, 3) promote *LOX* and *LOXL1-4* expression in human trabecular meshwork (sponge-like connective tissue located in the proximal side of the eye) cells *via* both Smad and non-Smad pathways (MAPK/JNK and AP-1) through TGFβR1 and TGFβR2 (Sethi *et al*., 2011). Additionally, the specific BMP antagonist gremlin induces *LOX* and *LOXL1-4* expression through constitutively active TGFβ/Smad and inducible MAPK/JNK signaling pathways, suggesting BMPs may at least partially mediate enzymatic collagen crosslinking (Sethi *et al*., 2013). In tendon, more work is needed to understand how the TGFβ family impacts LOX and LOXL1-4.

### Glycoprotein interactions

The KGHR sequence is a highly conserved amino acid binding sequence unique to fibrillar collagen and is involved in collagen crosslinking (Rodriguez-Pascual and Slatter, 2016; Sekiya *et al*., 2011). Currently, the glycoproteins TSP-1 and fibromodulin are the best understood ECM proteins that specifically interact with the KGHR binding motif in collagen. *Thbs1*-null mice experience altered crosslinking in the dermis, less abundant levels of proLOX and mature LOX, and an increased ratio of mature LOX to proLOX (Rosini *et al*., 2018). Moreover, TSP-1 inhibits maturation of proLOX through interactions with BMP-1 and collagen type I-III *in vitro* and binds both intracellular and extracellular collagen through the KGHR binding motif (Kalamajski *et al*., 2016; Rosini *et al*., 2018). TSP-1 is a particularly intriguing indirect regulator of enzymatic collagen crosslinking because it regulates fibrillogenesis at the intracellular procollagen level as well as at the extracellular fibrillar collagen level, and through regulation of LOX maturation. Interestingly, TSP-1 is also a well-documented regulator of the TGFβ superfamily in fibrotic diseases (Murphy-Ullrich and Suto, 2018). Further investigation of the various roles of TSP-1 in tendon development and function is warranted.

Fibromodulin deficient mice (Fmod-null) have altered tendons (mechanically weaker and morphologically altered) that exhibit increased LOX-induced mature crosslinks within collagen type I C-telopeptide domains, but LOX itself does not appear to be affected through post-translational modifications (Kalamajski *et al*., 2014). Fibromodulin, a SLRP, appears to alter the crosslinking behavior through interactions with LOX at the surface molecules of growing fibrils. This does not directly affect LOX quantity or processing, emphasizing the importance of not only stability and quantity of crosslinks but also location on the collagen fibril (Kalamajski *et al*., 2014; 2016).

Together, these studies on TSP-1 and fibromodulin highlight the importance of further investigating the interactions between glycoproteins that bind to the surface of collagen fibrils (*e.g*., the KGHR motif) and collagen crosslinking. The dual binding of these glycoproteins to fibrillar surface domains demonstrates that LOX-mediated crosslinking is modulated through a variety of interactions that are not yet well understood in tendon.

### Chaperone proteins

One study on CypB (an ER-resident chaperone protein)-null mice, used as a model for studying recessive osteogenesis imperfecta, showed that tail tendons exhibit poorly organized and abnormal morphology. CypB contributes, but does not appear to be essential, to the P3H complex that regulates LH isoforms and procollagen crosslinking (Terajima *et al*., 2016). Inhibiting CypB in tendon appears to impair fibrillogenesis and collagen morphology by altering lysine hydroxylation of collagen molecules, potentially through modulation of LH1-3 selectivity and activity, alteration of the P3H complex, or modification of collagen-SLRP interactions (Kalamajski *et al*., 2014; Robinson *et al*., 2005). The role of chaperone proteins in the regulation of enzymatic crosslinking needs to be further explored in tendon.

### Estrogen

Risk of Achilles tendon rupture may be reduced in females compared to males, suggesting an unknown protective mechanism for females (Sarver *et al*., 2017). Pardes *et al*. (2016) identified increased material properties (linear and dynamic moduli), decreased viscoelastic properties (hysteresis, percent relaxation), and decreased failure load in female rat Achilles tendons, suggesting female tendons may have more resistance to deformation under load and more efficient energy transfer. Comparing male and female tendons, no differences in tendon organization, cell shape, cellularity, proteoglycan content, or muscle fiber type were observed, indicating an unknown mechanism driving these disparities (Pardes *et al*., 2016). Minimal information is available on sex-related crosslinking differences, but a potential mechanism involved in these sex-related mechanical differences may include sex hormones, such as estrogen. An *in vitro* study found that estrogen can impact LOX levels in ligament cells (Lee *et al*., 2015). Fibroblasts derived from human ACLs, seeded in fibrin gels, and treated with peak physiologically relevant estrogen levels (*i.e*., 3–4 d before ovulation) have reduced mechanical properties (ultimate tensile stress, modulus) and LOX activity, but no changes in collagen content. Interestingly, LOX activity is more severely inhibited with estrogen treatment than LOX mRNA, suggesting estrogen may affect LOX primarily post-translationally (Lee *et al*., 2015).

When comparing the results of these studies, it is important to note that while Achilles tendons in females appear to be more protected from injury than males, females have an increased risk of ACL rupture, especially during menstruation (Arendt and Dick, 1995; Sarver *et al*., 2017). Unique sex-related differences between ligaments (*e.g*., ACL) and tendons (*e.g*., Achilles) and rates of injury may be attributed to the different mechanical roles of the tissues, but also several other confounding factors (Q-angle, age, sport being played, *etc*.). Together, these results suggest LOX may be partially controlled post-translationally by estrogen and justifies further investigation into the effects of estrogen on collagen crosslinking (*e.g*., LOX, LH, BMP-1, *etc*.) within tendon.

## Tissue engineering applications of LOX

Manipulation of LOX and associated proteins for tendon tissue engineering applications shows promise and recent interest. A 2020 patent titled “Enhancing Tissue Mechanical Properties” was filed (patent number US20200268935A1; Web ref. 1). This patent proposed to enhance tissue mechanical properties by increasing LOX or LOXL1-4 activity using techniques including mechanical stimulation to treat injuries and related musculoskeletal conditions (Web ref. 1).

Although currently less investigated for tendon tissue engineering applications, scaffold-free approaches have been attempted to regulate LOX-mediated collagen crosslinking for skin and cartilage in order to improve tissue formation. A scaffold-free 3D skin model was constructed from cultured fibroblasts derived from human stromal tissues and, when treated with TGFβ1 and BAPN (to inhibit LOX and LOXL4), appeared to be less fibrogenic compared to TGFβ1-only treated controls (Huang *et al*., 2019). Thus, inhibition of LOX and/or associated proteins within tissues that experience post-injury fibrosis (*e.g*., excessive amount of collagen often leading to scar formation), such as tendon, has the potential to facilitate restoration of pre-injury mechanical function. Similarly, injection of BAPN combined with controlled exercise has been used to improve fiber alignment and reduce the risk of reinjury in adult horse tendons (Dyson, 2004), potentially through a similar mechanism relating LOX/LOXL4 and antifibrogenic phenotypes seen in the fibrotic skin models (Huang *et al*., 2019). For cartilage tissue engineering applications, LOXL2 has been used in combination with TGFβ1 and chondroitinase-ABC to form 3D neocartilage from expanded articular chondrocytes (Kwon *et al*., 2019). This novel strategy combining manipulation of enzymatic crosslinkers using exogenous LOXL2 supplementation appears to be a promising technique for engineering more physiologically relevant cartilage constructs (Kwon *et al*., 2019). To the authors’ knowledge, the enhancement of exogenous LOX activity has not been performed towards engineered tendon formation *in vitro*.

LOX and associated collagen crosslinks have been used in tissue engineering applications but have yet to be fully explored for tendon applications. Further understanding of the effect of LOX on mechanical properties and LOX regulation will prove beneficial to develop novel tendon tissue engineering strategies.

## Non-enzymatic crosslinking

Differently from enzymatic crosslinks, non-enzymatic crosslinks typically form after adolescence and accumulate throughout adulthood. It was suggested that slow turnover of collagen in tendon allows for non-enzymatic crosslinking to accumulate (Avery and Bailey, 2005; Mentink *et al*., 2002), with the most prevalent type of non-enzymatic crosslinks being AGEs (see Table 2 for a summary on recent studies investigating AGE formation and impacts on tendon). AGEs form through Maillard reactions when reducing sugars, such as glucose, react with proteins (Fig. 2b) (Ahmed *et al*., 2012). The Maillard reaction is the same reaction that causes bread and meat to brown under high heat and tendons that have accumulated a large amount of AGEs appear yellow due to this reaction (Lee and Veres, 2019). Glucosepane is an AGE derived from D-glucose and presents lysine-arginine covalent links. Glucosepane is the most commonly found AGE in tendons, occurring 1,000 times more frequently than other AGEs (Jost *et al*., 2018). Pentosidine, a similar AGE, commonly used as a quantitative biomarker for AGEs, is formed from ribose and presents lysine-arginine links.

Originally, AGEs were thought to result in spontaneous and non-specific crosslinking between helical regions of collagen molecules, which contrasts with mature enzymatic crosslinks that primarily form at the telopeptidyl region of collagen molecules in tendon (Eyre *et al*., 2008). However, a recent study found that AGE-mediated crosslinks occur on the same helical Hyl sites in the collagen molecule as enzymatic crosslinks, suggesting that AGEs may impair the enzymatic crosslinking profile through steric interactions and have the potential to impact tissue mechanical properties (Hudson *et al*., 2018). These findings focused on Hyl-derived glycations and only minorly examined lysine-derived glycations (because rat tail tendons have an appreciably lower concentration of mature lysine-derived crosslinks). Therefore, understanding the location of both Hyl- and lysine-glycated crosslinks and how they interact with enzymatic crosslinking in tendon needs further study.

### AGE accumulation due to aging

AGEs increase as a function of age and may play a role in the age-related changes of tendon mechanical properties. Biochemical analysis of old (ages 67 ± 3 years) and young (ages 27 ± 2 years) human patellar tendons found aged tendons have decreased total collagen level, increased levels of both HP and LP enzymatic crosslinks, and increased pentosidine level (as a representative AGE), compared to young tendons (Couppé *et al*., 2009). The increased crosslinking seen in aged patellar tendon was associated with a decreased maximum force; however, other properties (strain, stiffness, stress, modulus, or CSA) were not affected (Couppé *et al*., 2009). In contrast, a different study using C57BL/6 mice tail tendons found that while immature crosslink levels decreases with age (resulting in a net decrease in total crosslinks), mature crosslinks (pyridinoline, glucosepane, pentosidine) and total lysine glycation levels (Fig. 4b) increase with age, suggesting that changes to both enzymatic and non-enzymatic crosslinks may contribute to tendon stiffening with age (Fig. 4c) (Stammers *et al*., 2020a). Together, these results justify further investigation of the specific changes of non-enzymatic crosslinks within tendon over time.

Several changes in structural (reduced tendon CSA and fibril diameter), cellular (decreased cell proliferation and stem/progenitor cell quantity), and mechanical properties (reduced modulus and strength), beyond just alterations to AGE-mediated crosslinks, occur in tendon during the aging process and have been recently reviewed in detail (Svensson *et al*., 2016). Briefly, few studies have specifically examined the relationship between aging and collagen deformation mechanisms (*i.e*., fiber sliding, helical rotation, *etc*.). One study explored the age-specific response of equine SDFTs, an energy-storing tendon, and found that aged tendons have a decreased ability to withstand fatigue loading (cyclic loading) due to aged fascicles being less capable of helical rotations (Thorpe *et al*., 2014). While the specific mechanisms culminating in impaired helical twist were not examined, other age-associated changes, such as age-related AGE accumulation and crosslinking (Stammers *et al*., 2020a), could account for the diminished mechanical response through interaction with the collagen matrix.

### AGE accumulation due to diabetes and diet

The slow turnover rate of collagen molecules within tendon makes it a tissue particularly susceptible to excess free glucose associated with hyperglycemia, as seen in diabetic patients, and it may induce excessive AGE accumulation at more dramatic rates than healthy counterparts (Brennan, 1989; Lee and Veres, 2019). Diabetic conditions are known to increase the elastic modulus in human Achilles tendon (Couppé *et al*., 2016). However, in diabetic mouse Achilles, supraspinatus, and patellar tendons stiffness decreases but the modulus is not affected (Connizzo *et al*., 2014). The underlying mechanisms driving these changes in mouse and human are unknown. Diabetic mice have decreased tail tendon collagen solubility compared to non-diabetic mice, suggesting that AGE accumulation may limit collagen remodeling and make the tendon more susceptible to microdamage accumulation (Babu *et al*., 2008). However, AGE accumulation is partially mitigated by a green tea treatment (Babu *et al*., 2008). Specifically, a green tea extract decreases Ehlrlich-positive material (*i.e*., tryptophan positive proteins), a marker of advanced glycation, and increases the tendon solubility of diabetic mice, suggesting hyperglycemic-induced glycation and AGEs can be therapeutically targeted.

AGE accumulation is also affected by the diet. Mice fed a normal diet with a large AGE content show higher levels of AGEs (pentosidine, carboxymethyllysine, methylglyoxal-derived hydroimidazolone) in both Achilles and tail tendons (with the Achilles having higher levels than the tail tendons) compared to mice fed a high-fat low-AGE content diet (Skovgaard *et al*., 2017). Mechanisms governing these tendon-specific responses to diet are not well understood but could be attributed to functionally distinct loading environments and collagen turnover. More specifically, previous work has identified increased AGE content and reduced collagen turnover in high strain, frequently injured tendons (equine SDFT) compared to low strain, infrequently injured tendons (equine common digital extensor tendon) (Thorpe *et al*., 2010b), reinforcing a potential relationship between loading environment and diet-induced AGE accumulation. Further study into the mechanisms governing tendon-specific diet-induced AGE accumulation and the potentially protective effects of diet will be needed.

### *In vitro* evaluation of AGE accumulation effects on tendon mechanics

Ribose and MGO are commonly used treatments that induce AGE formation and can be used to study the effects of non-enzymatic crosslinking on tendon. These treatments are effective at accelerating and simulating AGE accumulation starting from glucose *in vitro* (normally a gradual process *in vivo*) in tendon.

#### Ribose-induced AGEs

Ribose-glycated rabbit Achilles tendons show increased mechanical properties (maximum stress, strain, Young’s modulus, stiffness) and pentosidine (as a representative AGE), but decreased total collagen content (Reddy, 2004). In another study, two-month-old mouse tail tendons treated with ribose demonstrated increased hydration levels and decreased transverse stiffness (Andriotis *et al*., 2019). Accordingly, as tissue hydration can affect mechanical properties (Ntim *et al*., 2005), AGE accumulation could be impacting tissue mechanics through changes to hydration levels. Further work using ribose treatment has shown that AGE crosslinking negatively affects the ability of collagen fibrils to respond to loading after the yield point and results in a mechanically inferior tissue (Lee and Veres, 2019). An 11 d-glycated rat tail tendon collagen fibril showed that the intermolecular spacing increased by 0.05 nm and the D-period length decreased by 0.04 nm (Gautieri *et al*., 2017). However, a possible confounding factor is that ribose models tend to use a supraphysiological ribose concentration, which may induce an excessive AGE crosslinking profile. Thus, conclusions from these models will need to be confirmed *in vivo* (Gautieri *et al*., 2017; Vicens-Zygmunt *et al*., 2015). Together, these studies suggest that ribose treatment to stimulate the formation of AGEs is viable and could be further utilized to study the impacts of non-enzymatic crosslinks upon multiscale tendon mechanics.

#### MGO-induced AGEs

MGO is a dicarbonyl derived as a metabolic by-product of glycolysis and forms AGEs at a quicker rate than *in vivo* glucose reactions. Although, similarly to ribose, excessive MGO treatments can be less physiologically relevant (Li *et al*., 2013; Schalkwijk and Stehouwer, 2020). Rat tail tendons with MGO-derived AGEs show diminished fiber sliding and increased fiber-stretch but no obvious fibril-level ultrastructural changes (Li *et al*., 2013). Furthermore, MGO-treatment affects the tendon biomechanics (reduced stress relaxation, increased yield stress and ultimate failure stress). These results may suggest that the tissue changes associated with AGE accumulation could be driven by alterations to fiber deformation mechanisms, such as fiber-fiber sliding. Further investigation using SAXS found that MGO-treated fibrils exhibited altered molecular deformation (the associated quarter-staggered helical conformation was more resistant to strain) when mechanically loaded compared to untreated controls (Fessel *et al*., 2014). An increase in fibril failure resistance was also identified by a diminished capacity for collagen molecule-molecule sliding within fibrils, an observation potentially driven by AGEs bonding between fibrils. Interestingly, these changes to fibril deformation mechanics did not correspond to changes in fibril modulus and, thus, lend further support to the previous findings that AGE-derived mechanical changes are likely driven by changes at a larger scale, such as fiber-fiber sliding (Fessel *et al*., 2014; Li *et al*., 2013). These findings, while highly informative, are challenged by a lack of understanding regarding the specific number of MGO-mediated crosslinks and their location within the tail tendons. However, MGO models appear to be an effective means for studying AGE accumulation in tendon.

#### Glutaraldehyde-induced AGEs

Glutaraldehyde is another potent inducer of non-enzymatic crosslinks. Gachon and Mesquida (2020) found that collagen fibrils from adult rat tail tendons treated with glutaraldehyde do not show normal strain-stiffening and strain-softening behavior (*i.e*., excessive crosslinking causes fibrils to continue stiffening through higher strains, resulting in a more brittle behavior during fracture), differently from untreated control fibrils that exhibit strain-stiffening and strain-softening (*i.e*., fibrils initially straighten in response to strain before the fibrils themselves undergo softening at higher strains). Notably, these findings contradict a similar work on rat tail tendons using MGO-derived crosslinks that suggests AGE accumulation impacts tissue mechanics through fiber-level mechanisms, not alterations to fibrils (Li *et al*., 2013). Disparities between these studies may be attributed to different methods to induce non-enzymatic crosslinks (*i.e*., glutaraldehyde *vs*. MGO) and mechanical testing platforms. However, it will be important to identify how different crosslinking mechanisms affect fibril and fiber-level mechanics in tendon.

#### Glucosepane-induced AGEs

The effects of glucosepane, the most abundant lysine-arginine-derived AGE, have been recently investigated using computational modeling. Nash *et al*. (2019) coupled experimental methods with a computational model. Glucosepane and hydration levels increase in human Achilles and tibialis anterior tendons over time. Using molecular dynamics simulations, intramolecular glucosepane was demonstrated to negatively alter collagen structure and ordered collagen packing, as well as to increase hydration (unbound water molecules) around collagen molecules (Nash *et al*., 2019). A different simulation found that glucosepane and DOGDIC (another AGE crosslink) accumulation potentially increase Young’s modulus at low-range strains (0–15 %). However, the effects vary depending on where along the collagen molecules the crosslinks occur (Collier *et al*., 2018). A similar study focusing on arginine-lysine MODIC, GODIC, and DOGDIC crosslinks showed that, differently from glucosepane, DOGDIC seems to not inhibit collagen organization through spatial interactions but rather facilitate ordered collagen packing (Nash *et al*., 2021). These findings could be attributed to the smaller structure of DOGDIC compared to glucosepane (*i.e*., glucosepane has a much larger and obstructive structure than DOGDIC) altering its binding characteristics. Experimentally derived observations to validate these models will be an important future direction as well as identifying the specific binding locations. Overall, these findings justify both further investigation of various AGEs in tendon and the development of new methods to study AGEs *in vivo*.

## Regulation of AGEs by mechanical loading

Mechanical loading and AGEs both have protective effects against collagen-remodeling enzymes (Paik *et al*., 2006; Wyatt *et al*., 2009; Zareian *et al*., 2010). However, more recent work investigating AGE crosslinking in tendon found that loading, when combined with AGE accumulation, increases enzymatic degradation rates (Bourne *et al*., 2014). Specifically, rat tail tendons treated with ribose for 3 (less crosslinked) or 7 d (more crosslinked) to induce AGE crosslinking and, then, exposed to tensile strains were both more susceptible to degradation from bacterial collagenases compared to controls (Bourne *et al*., 2014). This observation could possibly be explained by the accumulation of AGEs disrupting the regular force-transferring mechanisms of collagen fibrils. The relationship between non-enzymatic crosslinking and loading was explored in mature rat tail tendons treated with sodium borohydride to fix the number of crosslinks and glycated lysine residues within the tissue (Stammers *et al*., 2020b). Sodium borohydride-fixed tendons stretched to 4 % strain exhibited an impaired mechanical response (reduced stress relaxation, increased failure stress, and reduced plastic deformation, *i.e*., being more brittle), demonstrating that dynamic crosslinking and glycation (glycations and crosslinks breaking and reforming in response to stimuli to attenuate mechanical load) are necessary for proper tissue function (Stammers *et al*., 2020b). Another study explored the effects of human life-long endurance running (as an extended loading environment model) and found that both young and aged runners show tendon hypertrophy and a 21 % decrease in AGE density, compared to aged controls (Couppé *et al*., 2014). The observed decreases in AGE content from endurance running could suggest a mechanoresponsive behavior in tendon AGE accumulation, perhaps relating to a relationship between long-term mechanical loading and dynamic crosslinking. These studies describe a complex relationship between non-enzymatic collagen crosslinks and mechanical loading that deserves further study.

Overall, non-enzymatic crosslinks, specifically AGEs, are physiologically relevant in tendon because of diabetes and aging. AGEs form through the relatively slow process of glycation that, when coupled with the slow turnover rate of collagen in tendon, leads to an accumulation of crosslinks during aging that affects tendon mechanical properties. AGE accumulation appears to impact collagen at the fiber and fibril levels, and conditions that induce excessive AGE formation are linked to negative tendon outcomes. Therefore, further investigation is needed to determine the specific mechanical effects of AGEs, how they interact with collagen molecules, and to identify mechanisms that may regulate AGE accumulation and removal.

## Conclusion

Enzymatic and non-enzymatic collagen crosslinking plays a role in regulating the mechanical function of tendon. Multiple collagen crosslinking enzymes (*e.g*., LOX and LOXL2-4) are needed for proper collagen crosslinking both during and after fibrillogenesis, which mediates the mechanical formation of tendon and may distinguish functionally distinct tendons (energy storing *vs*. positional). However, the mechanisms governing enzymatic crosslinking are not well understood and require further investigation. Potential regulators of enzyme production at the cellular level may include mechanical stimuli, mechanotransducive cell signaling pathways, sex hormones, TGFβ family, hypoxia, and interactions with intracellular or extracellular proteins.

AGE crosslinks derived from non-enzymatic interactions resulting from free glucose impact tendon function. AGEs, which accumulate during the normal aging process, can be accelerated under diabetic conditions and may also be affected by diet. The specific consequences of AGE accumulation are not entirely understood but may alter both fiber and fibril behavior. An improved understanding of the exact location and process through which these crosslinks form is needed. Overall, enzymatic and non-enzymatic crosslinks affect tendon biomechanics and, thus, a better understanding of their regulators can advance future clinical treatments for tendon disease, injury, and age-related conditions.

## Figures and Tables

**Table 1.** Recent studies exploring potential regulators of enzymatic collagen crosslinking in tendon, ligament, and related tissues.

Potential regulator	Species/tissue	Age/time point	Treatment	Evaluation	Major findings	Source
**Mechanical stimuli**	Chick tendon	HH 28-HH 45, tendon explant cultured for 48 h	Paralysis, hypermotility, BAPN	Mechanical testing, *LOX/LOXL* expression and activity	Paralysis (unloading) decreases modulus and LOX activity, LOXL isoforms respond uniquely to mechanical loading	Pan *et al.,* 2018
	Human-derived periodontal ligament cells	24 or 48 h culture	3 % or 10 % mechanical stretch	RT-PCR, fluorescence imaging	3 % stretch promotes LOX and collagen production whereas 10 % stretch upregulates *MMP2* expression with no changes to LOX activity	Chen *et al.,* 2016
	Human-derived periodontal ligament cells, rat periodontal ligament	12 or 24 h culture, 3 d of treatment	Compressive loading, excessive occlusal loading	LOX/LH2/collagen gene expression, tissue histology	Low loading increases LOX/LH2 expression, excessive occlusal loading increases collagen maturation and LOX/LH2 expression	Kaku *et al.,* 2016
	Rat tail tendon, mouse tail tendon	14–18 weeks (rat), 10–39 weeks (mouse)	PIEZO1 gain (or loss) of function	Mechanical testing, calcium imaging, crosslink analyses	PIEZO1 affects enzymatic crosslinking density and crosslinking appears to be mechanosensitive	Passini *et al.,* 2021
**Hypoxia**	Bovine patellar tendon and articular cartilage	4–8 weeks old bovine tissue explant cultured for 4 weeks	Hypoxia, exogenous LOXL2	Mechanical testing, LOX expression, crosslink analyses	Increased number of mature crosslinks, increased *LOX* expression (evaluated in articular cartilage only), and increased modulus with hypoxia and LOXL2 treatment	Makris *et al.,* 2014
**TGFβ signaling**	Human trabecular meshwork cells	24 h treatment in culture	Exogenous TGFβ, inhibitors of TGFβ signaling	RT-PCR, Western blot	TGFβ1-3 isoforms promote *LOX* and *LOXL1-4* expression through canonical and non-canonical TGFβ signaling pathways	Sethi *et al.,* 2011
	Human trabecular meshwork cells	24 h treatment in culture	BMP antagonist, inhibitors of TGFβ signaling	*LOX/LOXL1-4* expression	BMP antagonist gremlin induces *LOX* and *LOXL* expression through TGFβ signaling	Sethi *et al.,* 2013
**Protein interactions**	Mouse dermis, human-derived fibroblasts	8 weeks old, 24–96 h culture	TSP-1 knockout	Solid-phase binding assays, fluorescence imaging	TSP-1 binds to intracellular and extracellular collagen and interacts with dermal LOX activity	Rosini *et al.,* 2018
	Mouse tail tendon	5 months old	Fibromodulin knockout	Solid phase proximity ligation assay, histology	Fibromodulin interacted with LOX at the surface of collagen fibrils	Kalamajski *et al.,* 2016
	Mouse tail tendon	2 months old	Cyp B knockout	TEM imaging, mass spectrometry, histology	Cyp B knockout impairs tendon fibrillogenesis; altered lysine hydroxylation	Terajima *et al.,* 2016
**Estrogen**	Human ACL-derived fibroblasts	20–24 year old fibroblasts cultured for 14 d	Exogenous estradiol, BAPN	Mechanical testing, hydroxyproline assay, LOX activity assay	Estrogen reduces tissue engineered construct mechanical properties and LOX activity	Lee *et al.,* 2015

**Table 2.** Recent studies on AGE formation and impacts in tendon.

Treatment	Species/tissue	Age/time point	Analysis	Major findings	Source
**Aging**	Human patellar tendon	67 ± 3 years, 27 ± 2 years	Mechanical testing, collagen crosslink analysis	Aged tendons show increased HP, LP, and pentosidine crosslinks and decreased maximum force	Couppé *et al,* 2009
**Diabetic conditions**	Mouse tail tendon	26 weeks old	HPLC for crosslink analysis, mass spectrometry	AGE-mediated crosslinks interact in the same Hyl domains as enzymatic crosslinks	Hudson *et al.,* 2018
	Human Achilles tendon	58–60 years old	Mechanical testing, TEM	Diabetic patients show increased Young’s modulus in tendon	Couppé *et al.,* 2016
	Rat tail tendon	9–10 weeks old	Collagen solubility, Ehrlich test, fluorescence imaging	Green tea treatment decreases markers of advanced glycation and increases collagen solubility	Babu *et al.,* 2008
**High AGE diet**	Rat Achilles and tail tendons	40 weeks old	Mass spectrometry, HPLC	High-AGE diet increases AGE content in Achilles and tail tendons, with Achilles having higher AGE levels than tail tendons	Skovgaard *et al.,* 2017
**Ribose**	Bovine tail tendons	18–30 months old	Mechanical testing, scanning calorimetry, SEM	Ribose treatment alters nanoscale collagen fibril deformation mechanisms in tendon	Lee and Veres, 2019
	Rat tail tendon	6 months old	Fluorescence imaging, enzyme susceptibility assay, mechanical testing	Ribose-treated and mechanically loaded tendons are more susceptible to collagenase activity	Bourne *et al.,* 2015
**MGO**	Rat tail tendon	3, 12, and 22 months old	Collagen solubility, SDS-PAGE, mass spectrometry	Glucosepane is the predominant AGE; MGO treatment increases tendon stability	Jost *et al.,* 2018
	Rat tail tendon	> 17 weeks old	Mechanical testing, multiphoton imaging	MGO treatment diminishes fiber-fiber sliding and increases fiber stretch	Li *et al.,* 2013
	Rat tail tendon	17–24 weeks old	Mechanical testing, SAXS	MGO treatment alters molecular collagen deformation mechanisms (increased fibril failure resistance and reduced molecular sliding within fibrils)	Fessel *et al.,* 2014
**Glutaraldehyde**	Rat tail tendon	Adult	Nanoindentation	Glutaraldehyde treatment results in a more brittle behavior during fibril fracture	Gachon and Mesquida, 2020
**Sodium borohydride**	Mouse tail tendon	11 weeks old	Mechanical testing, HPLC mass spectrometry	Reduction with sodium borohydride decreases stress relaxation and plastic deformation and increases failure stress	Stammers *et al.,* 2020

## List of Abbreviations

ACL: anterior cruciate ligament
AGE: advanced glycation end product
AP-1: activating protein-1
BAPN: β-aminopropionitrile
BMP-1: bone morphogenetic protein-1
Col1a1: collagen type I alpha 1 chain
Col3a1: collagen type III alpha 1 chain
CRL: cytokine receptor-like
CSA: cross-sectional area
CypB: cyclophilin B
DOGDIC: 3-deoxyglucosone-derived imidazolium
ECM: extracellular matrix
ER: endoplasmic reticulum
GODIC: glyoxal-derived imidazolium
HH: Hamburger-Hamilton
HIF: hypoxia inducible factor
HP: hydroxylysyl pyridinoline
hPDL: human periodontal ligament
HPLC: high-performance liquid chromatography
Hyl: hydroxylysine
JNK: c-Jun N-terminal kinases
LH: lysyl hydroxylase
LOX: lysyl oxidase
LOXL: LOX-like protein
LOX-PP: LOX pro-peptide
LP: lysyl pyridinoline
LTQ: lysyltyrosine quinone
MAPK: mitogen-activated protein kinase
MCL: medial collateral ligament
MCP: 2-mercaptopyridine-N-oxide
MGO: metabolite methylglyoxal
MMP: matrix metalloproteinase
MODIC: methylglyoxal-derived imidazolium
P3H: prolyl-3-hydroxylase
P4HA: prolyl 4-hydroxylase subunit alpha
PCP: procollagen-C-proteinase
PDI: protein disulfide isomerase
PDL: periodontal ligament
PIEZO1: Piezo type mechanosensitive ion channel component 1
PLOD: procollagen lysyl hydroxylase
Pyr: pyridinoline
RT-PCR: reverse transcription polymerase chain reaction
SAXS: small-angle X-ray scattering
SDFT: superficial digital flexor tendon
SDS-PAGE: sodium dodecyl sulfatepolyacrylamide gel electrophoresis
SEM: scanning electron microscopy
SLRP: small leucine-rich proteoglycan
SNAIL: snail
SOX9: SRY-box transcription factor 9
TCP: trans-2-phenylcylopropylamine
TEM: transmission electron microscopy
TG: transglutaminase
TGFβ: transforming growth factor beta
TGFβR: TGFβ receptor
Thbs1: thrombospondin-1 gene
TIMP: tissue inhibitor of MMP
TSP-1: thrombospondin-1
